# Fighting resistance with redundancy: a path forward for treating antimicrobial-resistant infections?

**DOI:** 10.1128/aac.00121-25

**Published:** 2025-03-14

**Authors:** Jose M. Munita, Pranita D. Tamma

**Affiliations:** 1 Instituto de Ciencias e Innovación en Medicina, Facultad de Medicina Clínica Alemana, Universidad del Desarrollo 441184 , Santiago, Chile; 2 Johns Hopkins University School of Medicine 1466, Baltimore, Maryland, USA; Houston Methodist Hospital and Weill Cornell Medical College, Houston, Texas, USA

**Keywords:** *Acinetobacter baumannii*, sulbactam-durlobactam, carbapenem-resistant

## Abstract

Carbapenem-resistant *Acinetobacter baumannii* (CRAB) remains a major threat, with high mortality and limited effective treatments. Sulbactam-durlobactam has emerged as a promising therapy against CRAB. Sulbactam-durlobactam was combined with imipenem-cilastatin in a clinical trial that led to its United States Food and Drug Administration approval. However, the additive benefit of imipenem remains uncertain. In a recent study (Antimicrob Agents Chemother 69:e01627-24, 2025, https://doi.org/10.1128/aac.01627-24), Veeraraghavan and colleagues provide convincing mechanistic evidence that adding imipenem to sulbactam-durlobactam enhances bacterial killing, likely through complementary inhibition of penicillin binding proteins, leveraging the concept of target redundancy.

## COMMENTARY

Carbapenem-resistant *Acinetobacter baumannii* (CRAB) is a formidable nosocomial pathogen that continues to challenge clinicians worldwide. For decades, the optimal treatment strategy for CRAB infections has remained elusive, resulting in significant mortality, particularly among critically ill patients (1, 2). However, a consistent finding across preclinical and clinical studies is that incorporating sulbactam into treatment regimens improves outcomes. Pharmacodynamic models have demonstrated that even in sulbactam-resistant CRAB infection models, high-dose sulbactam leads to a significant reduction in bacterial load (3–5). While clinical trials investigating sulbactam-based regimens for CRAB infections have not reliably resulted in improved patient outcomes, numerically lower mortality rates in the sulbactam-treated groups have been consistently reported, suggesting a potential benefit of sulbactam-based therapies (6–10).

Sulbactam, a penicillin derivative, efficiently inhibits CRAB penicillin-binding proteins (PBP) PBP1a, PBP1b, and PBP3 (11). However, its activity is significantly compromised by various β-lactamases frequently produced by *A. baumannii*, preventing it from reaching its PBP targets. These include Class A TEM-1 enzymes, Class C *Acinetobacter*-derived cephalosporinases, and Class D OXA carbapenemases (e.g., OXA-23, OXA-51) (12, 13).

The recent approval of sulbactam-durlobactam for clinical use marked a significant advancement, providing a valuable therapeutic option against CRAB. Durlobactam, a diazabicyclooctane β-lactamase inhibitor, effectively preserves sulbactam’s activity by inhibiting the aforementioned Ambler Class A, C, and D enzymes (14, 15). The pivotal clinical trial that led to sulbactam-durlobactam’s approval by the United States Food and Drug Administration evaluated its efficacy against severe CRAB infections as compared with colistin therapy (10). To ensure coverage against polymicrobial infections, both study arms also included imipenem–cilastatin. As a result, while clinical data supporting sulbactam-durlobactam’s use for CRAB infections were obtained in the presence of imipenem, the potential additive benefit of imipenem remains uncertain.

Veeraraghavan and colleagues provide valuable insights through meticulously designed experiments investigating the incremental benefit of sulbactam–durlobactam in the presence of imipenem (16). They hypothesized that the combination of two β-lactams with distinct targets — PBP1a, 1b, and 3 for sulbactam, and PBP2 for imipenem — enhance bacterial killing through the simultaneous deactivation of multiple PBPs. Previous *in vitro* studies showed that combining sulbactam–durlobactam with imipenem or meropenem leads to a one- to two-fold reduction in sulbactam minimum inhibitory concentrations (MICs) compared with sulbactam–durlobactam alone, forming the basis for this theory (17, 18). However, in the absence of mechanistic validation, these findings remained exploratory — until now.

The authors conducted time–kill assays demonstrating that the combination of sulbactam–durlobactam and imipenem consistently achieved greater than a 2-log CFU/mL reduction in CRAB bacterial load at 8 h (16). In contrast, sulbactam–durlobactam alone failed to achieve a 2-log reduction in any of the tested CRAB isolates, with four of the five unable to reach a 1-log reduction. Notably, while sulbactam–durlobactam reduced the bacterial load of both carbapenem-susceptible *A. baumannii* isolates by over 1-log, adding imipenem further enhanced killing by an additional log. This latter finding highlights the important role of β-lactamases commonly produced by even carbapenem-susceptible *A. baumannii* (e.g., TEM, ADC) in diminishing sulbactam’s activity.

Molecular docking analyses were performed to further elucidate the mechanisms underlying this synergy. Results confirmed the distinct affinities of sulbactam and imipenem for PBP3 and PBP2, respectively, while also demonstrating their lower affinity for PBP1a. These findings support the concept of enhanced bacterial killing through complementary target redundancy against *A. baumannii* PBPs. Furthermore, docking simulations confirmed durlobactam’s potent inhibition of OXA-23 and OXA-51 — carbapenemases commonly produced by CRAB isolates (2) — thereby limiting the enzymatic degradation of sulbactam and imipenem.

The concept of multiple target redundancy — particularly differential PBP inhibition — as a strategy to enhance bacterial killing has been explored in other bacterial pathogens (19). A notable example is *Enterococcus faecalis*, which exhibits natural tolerance to β-lactam antibiotics due to the production of the low affinity PBP4/5 (20, 21). Early *in vitro* studies suggested that combining ampicillin with cefotaxime enhanced bacterial killing compared with either agent alone (22, 23). This synergistic effect was later confirmed in clinical studies of patients with *E. faecalis* infective endocarditis (24). While the precise mechanism underlying this synergy remains unclear, the leading explanation is differential PBP saturation. Specifically, ampicillin partially inhibits PBP4/5, while cefotaxime fully saturates non-essential PBPs 2 and 3, creating a disruption in cell wall synthesis, ultimately leading to bacterial death.

Target redundancy may be a critical strategy to explore as bacteria can readily modify antibacterial targets, rendering essential drugs inactive. As an example, two compounds, aztreonam-avibactam and cefiderocol, are the preferred antibiotics for NDM-producing *Escherichia coli* infections (25), with both agents primarily inhibiting PBP3. Unfortunately, high-risk clones of *E. coli* with modified PBP3 enzymes exhibiting low affinity towards aztreonam and cefiderocol have emerged internationally (26). PBP3 resistance is primarily mediated by variants with four amino acid insertions that result in reduced accessibility to the active transpeptidase pocket (27, 28). The emergence of NDM-producing *E. coli* resistant to both preferred β-lactam agents highlights the hazards of relying on a single PBP target for antimicrobial activity — particularly against highly drug-resistant bacteria.

With CRAB infections carrying mortality rates exceeding 30% (1), the study by Veeraraghavan and colleagues highlights the potential benefit of adding imipenem to sulbactam–durlobactam therapy. However, a number of uncertainties remain regarding the risk-benefit ratio of this strategy. It is unclear whether the combination needs to be continued throughout the entire therapy or if a short initial course to increase bacterial killing would be sufficient to provide sustained advantages. This is particularly relevant given the logistical challenges associated with this treatment strategy. Furthermore, while existing data have not signaled adverse events with high-dose ampicillin–sulbactam plus a carbapenem (29, 30), the safety profile of sulbactam–durlobactam in combination with imipenem-cilastatin requires further investigation. Overall, this study marks an important advancement in the treatment of CRAB infections and emphasizes the role of PBP target redundancy in driving synergistic bactericidal activity.
